# Supplementary figures and images for: Spatial-temporal variability of Mytilus galloprovincialis Lamarck 1819 populations and their accumulated sediment in northern Portugal
Source: PeerJ. 2021 Jun 22;9:e11499. doi: 10.7717/peerj.11499 (PMC8231337; doi:10.7717/peerj.11499)

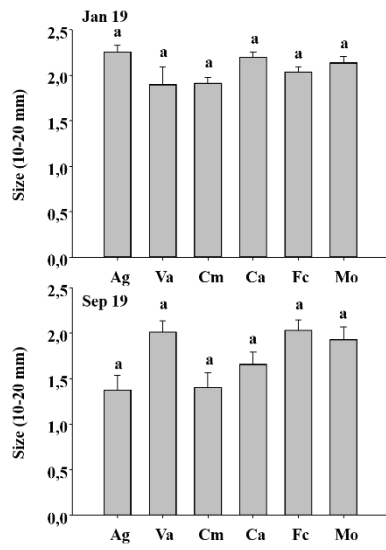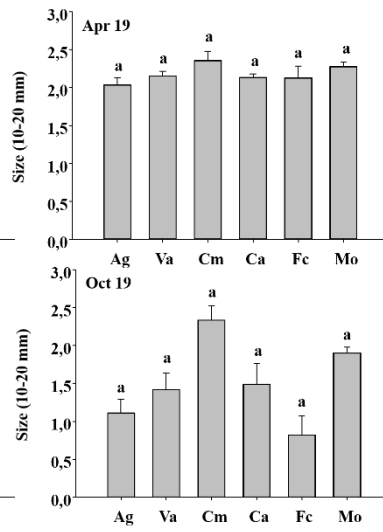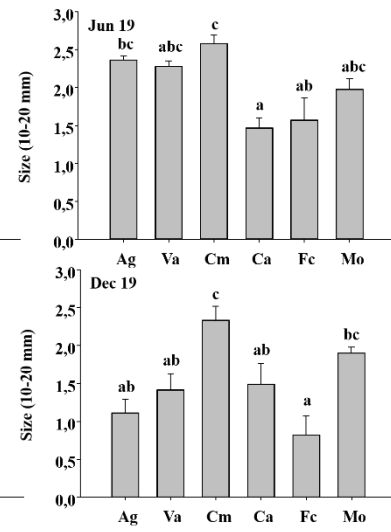

Supplement: Supplemental Information 1 — Mean values (+ SE) of size class in cm. Ag, Aguda; Va, Valadares; Cm, Cabo do Mundo; Ca, Carreço; Fc, Forte do Cão; Mo, Moledo. Different letters indicate significant differences between shores (P < 0.01) as detected by SNK test. [file peerj-09-11499-s001.pdf]

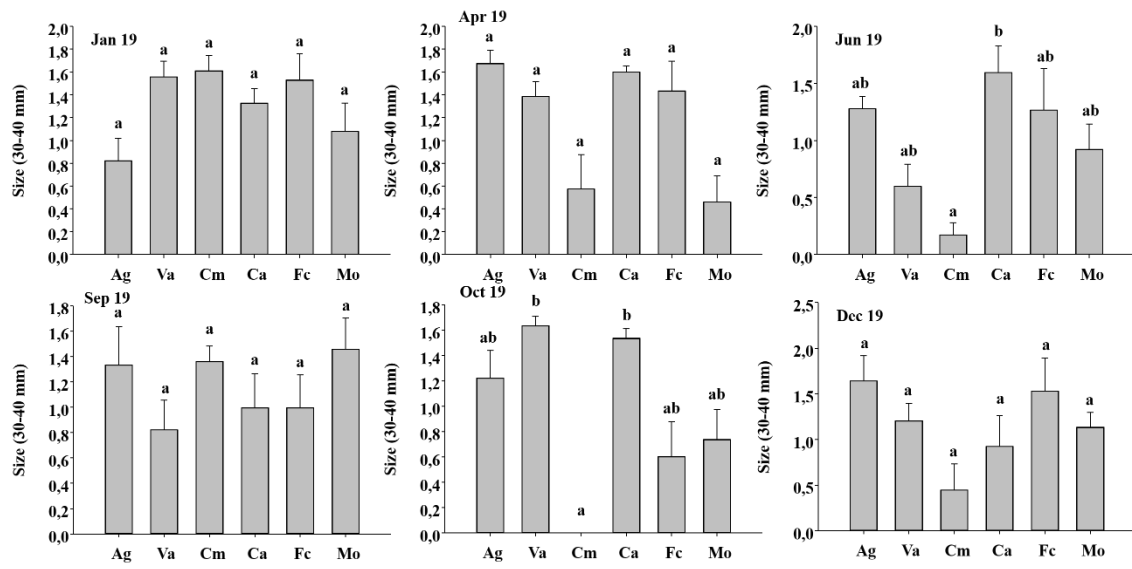

Supplement: Supplemental Information 2 — Mean values (+ SE) of size class in cm. Ag, Aguda; Va, Valadares; Cm, Cabo do Mundo; Ca, Carreço; Fc, Forte do Cão; Mo, Moledo. Different letters indicate significant differences between shores (P < 0.01) as detected by SNK test. [file peerj-09-11499-s002.pdf]

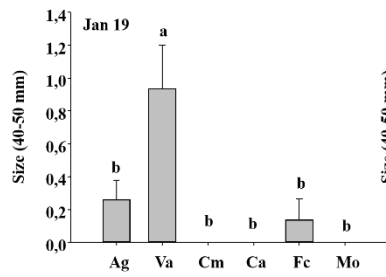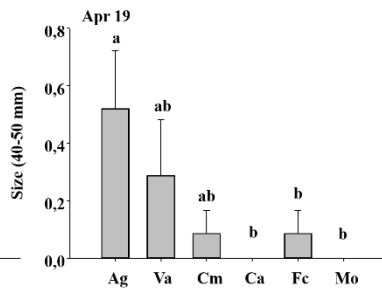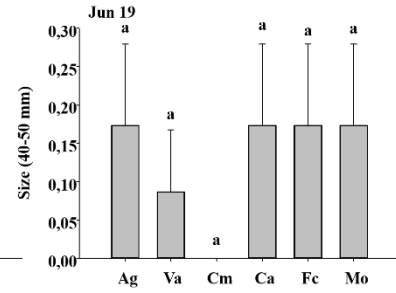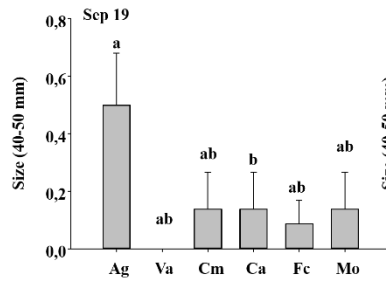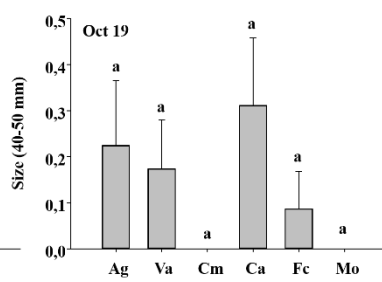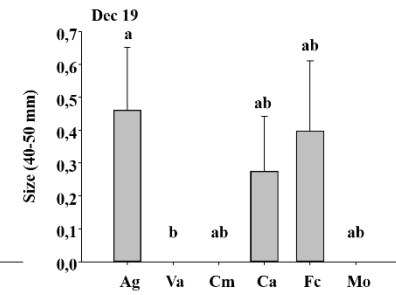

Supplement: Supplemental Information 3 — Mean values (+ SE) of size class in cm. Ag, Aguda; Va, Valadares; Cm, Cabo do Mundo; Ca, Carreço; Fc, Forte do Cão; Mo, Moledo. Different letters indicate significant differences between shores (P < 0.01) as detected by SNK test. [file peerj-09-11499-s003.pdf]

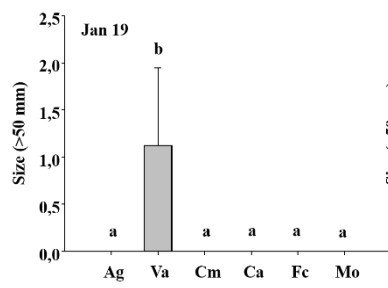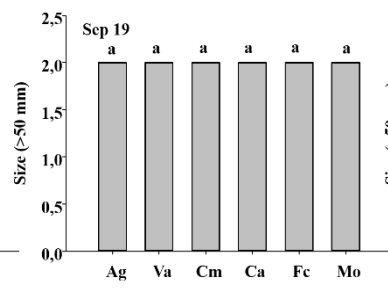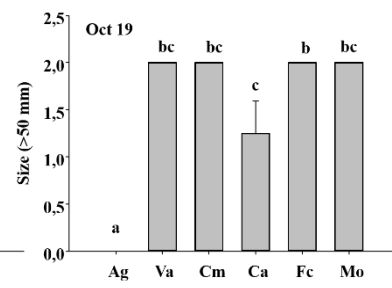

Supplement: Supplemental Information 4 — Mean values (+ SE) of size class in cm. Ag, Aguda; Va, Valadares; Cm, Cabo do Mundo; Ca, Carreço; Fc, Forte do Cão; Mo, Moledo. Different letters indicate significant differences between shores (P < 0.01) as detected by SNK test. [file peerj-09-11499-s004.pdf]
